# Photocrosslinkable Cellulose Derivatives for the Manufacturing of All-Cellulose-Based Architectures

**DOI:** 10.3390/polym16010009

**Published:** 2023-12-19

**Authors:** Maximilian Rothammer, Cordt Zollfrank

**Affiliations:** Chair for Biogenic Polymers, Technical University of Munich, Schulgasse 16, 94315 Straubing, Germany; maximilian.rothammer@tum.de

**Keywords:** cellulose derivative, photocrosslinking, UV curing, cellulose regeneration, bio-based photoresist, biomaterial, esterification

## Abstract

Replacing petroleum-based polymers with biopolymers such as polysaccharides is essential for protecting our environment by saving fossil resources. A research field that can benefit from the application of more sustainable and renewable materials is photochemistry. Therefore, cellulose-based photoresists that could be photocrosslinked via UV irradiation (λ = 254 nm and λ = 365 nm) were developed. These biogenic polymers enable the manufacturing of sustainable coatings, even with imprinted microstructures, and cellulose-based bulk materials. Thus, herein, cellulose was functionalized with organic compounds containing carbon double bonds to introduce photocrosslinkable side groups directly onto the cellulose backbone. Therefore, unsaturated anhydrides such as methacrylic acid anhydride and unsaturated and polyunsaturated carboxylic acids such as linoleic acid were utilized. Additionally, these cellulose derivatives were modified with acetate or tosylate groups to generate cellulose-based polymers, which are soluble in organic solvents, making them suitable for multiple processing methods, such as casting, printing and coating. The photocurable resist was basically composed of the UV-crosslinkable biopolymer, an appropriate solvent and, if necessary, a photoinitiator. Moreover, these bio-based photoresists were UV-crosslinkable in the liquid and solid states after the removal of the solvent. Further, the manufactured cellulose-based architectures, even the bulk structures, could be entirely regenerated into pure cellulose devices via a sodium methoxide treatment.

## 1. Introduction

In the view of ever-accelerating climate change [1,2,3], it is imperative to take countermeasures to preserve our planet for future generations. An important contribution to this is to conserve fossil resources. Nevertheless, the industry is still consuming a lot of mineral oil to produce educts for pharmaceutical compounds, cosmetics, plastics and even food [4,5]. These processes consume huge amounts of energy and produce large quantities of waste at the same time. Therefore, a more sustainable alternative to fabricating polymers is the replacement of fossil resources with the use of renewable ones. Consequently, polysaccharides are promising raw materials, as cellulose and chitin are among the most abundant biopolymers worldwide [6,7,8,9]. Cellulose is also characterized by other advantageous properties, such as biodegradability, non-toxicity, biocompatibility, inexpensiveness and a high chemical modification capacity [6,8,10,11].

Based on our previous research [12,13,14], which demonstrated that methacrylated polysaccharides and their monomers are suitable for additive manufacturing applications such as direct laser writing via two-photon absorption, we here expand, within this green chemistry approach, the range of bio-based materials using various photocrosslinkable cellulose derivatives. Therefore, in addition to cellulose diacetate (CDA), tosyl cellulose (TSC) was employed as a further educt, as it also exhibits an appropriate solubility in common organic solvents. Both educts were functionalized with various unsaturated and polyunsaturated carboxylic acids or anhydrides, which are required for a UV-induced photopolymerization reaction. To expand the existing class of sustainable photocrosslinkable polysaccharides, renewable and biogenic fatty acids such as erucic acid, oleic acid and linoleic acid, as well as further olefinic acids, namely 4-pentenoic acid and 3-butenoic acid, which can also be derived from bio-sourced educts [15,16], were utilized for the modification of the cellulose derivatives. Thus, this contributes to covering the growing demand for sustainable and bio-based chemical products with small carbon footprints [17]. Especially in photochemistry applications or additive manufacturing techniques via irradiation curing, there is an opportunity to provide new bio-based materials with only minor adjustments [12]. Consequently, this combines the benefits of additive manufacturing, such as rapid prototyping, high flexibility and the fabrication of complex geometries by applying innovative biomaterials based on renewable resources, that are omnipresent and available at a low cost [18,19].

Since cellulose has revealed a strong hydrogen network, pure cellulose is not suitable for many manufacturing methods, such as thermoplastic extrusion-based 3D printing, due to the decomposition that occurs before the melting point is reached [20,21]. In order to overcome this limitation, many applications for generating cellulosic architectures are based on solutions with low cellulose concentrations or even suspensions [22,23]. A very common path to producing cellulose-based structures via 3D printing utilizes cellulose derivatives in aqueous solutions, such as methacrylated carboxymethyl cellulose or hydroxypropyl cellulose [24,25]. Regarding the low cellulose concentrations and the large amounts of water, which has to be removed, for instance, via freeze-drying, within these hydrogels, the achieved devices are characterized by low density and limited dimensional stability [23]. To overcome these drawbacks, the derivatives presented in this work are soluble in several organic solvents, such as acetone, which is an inexpensive solvent that evaporates quickly due to its high vapor pressure [23,26]. This allows for layer-by-layer manufacturing of robust devices that can be cured with UV light. The olefinic cellulose derivatives synthesized in this report are suitable for multiple application methods, such as the coating, printing and even casting of bulk architectures. In addition, patterns down to the micrometer range can easily be transferred into cellulose-based films during photocrosslinking. Therefore, the applied photoresists consisted of cellulose derivatives dissolved in an organic solvent and, if necessary or in order to reduce the curing time, a photoinitiator. Upon UV irradiation, the photoinitiator was cleaved into free radicals, which induced the crosslinking of the cellulose chains by forming covalent bonds [12]. The feasibility of UV-induced crosslinking of sorbic cellulose diacetate without the addition of a photoinitiator has been recently reported [11]. Since crosslinking without a photoinitiator requires more time, all of the photoresists presented here contained around 2.5 wt.% initiator. Moreover, these polysaccharide-based photoresists can be used as coatings, as self-standing films with imprinted micropatterns or as robust bulk architectures. In addition, we have developed a process that makes it possible to entirely regenerate cellulose-derivative-based structures into pure cellulose, even the bulk structures, apparently, without changing their stability or shape. The regeneration process of the cellulose-derivative-based structures into pure cellulose via a sodium methoxide treatment aims for potential biodegradability.

## 2. Results and Discussion

### 2.1. Chemical Modification of the Cellulose Derivatives

**Figure 1:**
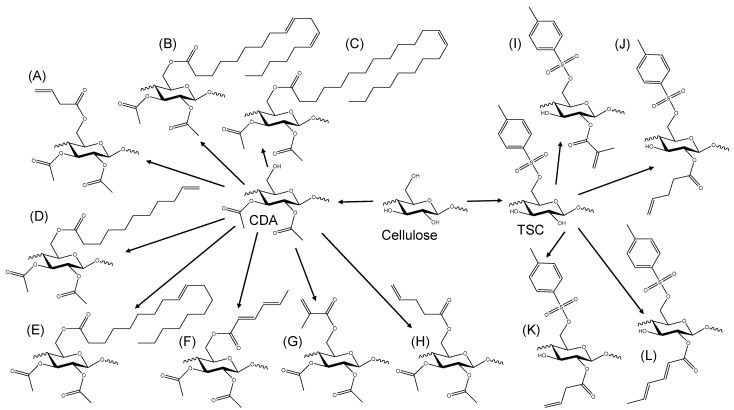
Schematic representation of the synthesis pathways starting from cellulose as a raw material, leading to the reactants CDA and TSC. This employed unsaturated or polyunsaturated carboxylic acids or anhydrides, resulting in various photocrosslinkable cellulose derivatives: (**A**) 3-butenoic CDA, (**B**) linoleic CDA, (**C**) erucic CDA, (**D**) 10-undecenoic CDA, (**E**) oleic CDA, (**F**) sorbic CDA, (**G**) methacrylic CDA, (**H**) 4-pentenoic CDA, (**I**) methacrylic TSC, (**J**) 4-pentenoic TSC, (**K**) 3-butenoic TSC and (**L**) sorbic TSC.

Cellulose is a linear homopolysaccharide that consists of β-1,4-glycosidic linked d-glucose units. Each monomer contains three reactive hydroxyl groups: one primary group at the C6 position and two secondary hydroxyl groups at the C2 and C3 positions [27,28]. Due to strong inter- and intramolecular hydrogen bonds, cellulose exhibits a semicrystalline supramolecular structure [6,28]. A consequence of the hydrogen bonds as well as of the supramolecular structure is the insolubility of cellulose in common organic solvents [27,29]. Thus, cellulose diacetate and tosyl cellulose were selected as raw materials for the functionalization, with carbon double bond-containing moieties (Figure 1). This would ensure the solubility of the photocrosslinkable biopolymers in various solvents. The synthesized derivatives were evaluated in detail considering the selected derivatives, while the remaining derivatives are characterized in the experimental section.

**Figure 2:**
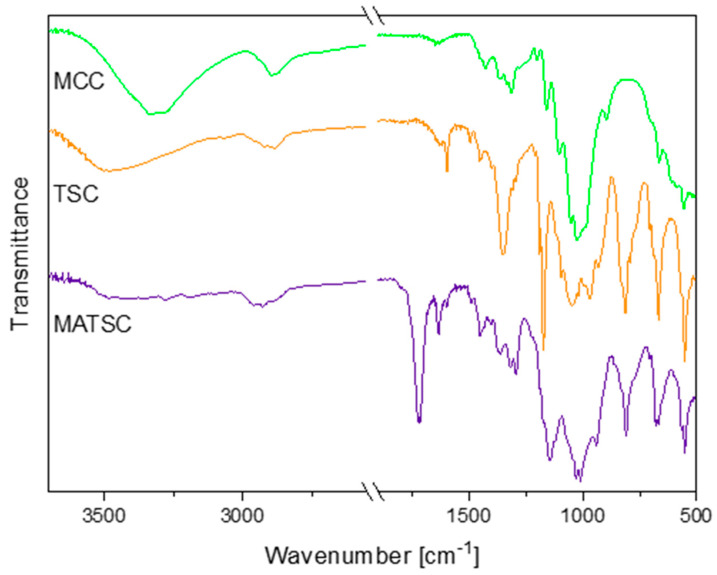
FTIR spectra of MCC, TSC and MATSC. The spectra are shifted vertically for better readability.

Fourier transform infrared spectroscopy (FTIR) analyses were performed on the educt microcrystalline cellulose (MCC), tosyl cellulose and functionalized TSC. This allowed for the verification of the tosylation of the MCC and the esterification of the TSC with methacrylic acid anhydride (Figure 2). The most significant FTIR absorption bands for unmodified and modified cellulose are discussed in the following. The cellulose was mainly characterized by the hydroxyl stretching vibration found at around 3334 cm^−1^, the CH stretching of CH_2_ groups at 2892 cm^−1^ and the C–O symmetrical stretching of primary alcohol at 1026 cm^−1^ [30,31]. The C–O–C antisymmetrical bridge, stretching to 1160 cm^−1^, and the β-glucosidic bonds between the sugar units of the cellulose, at 894 cm^−1^, were clearly recognizable [30,32].

Additionally, the signals of the sulfonic acid ester at 1352 cm^−1^, 1173 cm^−1^ and 812 cm^−1^ and the absorption bands of the aromatic ring at 3065 cm^−1^, 1597 cm^−1^, 1495 cm^−1^ and 1456 cm^−1^ confirmed the successful tosylation of the cellulose [29,31,33]. Thereby, the signal at 1352 cm^−1^ refers to the asymmetrical stretching of the –SO_2_– group, while the band at 1173 cm^−1^ has been assigned to the symmetrical one [34,35]. The absorption band at 812 cm^−1^ represented the S–O–C bond stretching vibration [29,34,36]. Moreover, the FTIR spectrum of the TSC indicated a strong shift of the hydroxyl stretching band, from 3334 cm^−1^ to 3500 cm^−1^.

The absorption band at 1722 cm^−1^ corresponded to the carbonyl C=O ester stretching vibration and verified the methacrylation of the tosyl cellulose (MATSC). Additionally, new absorption bands arose at 1633 cm^−1^ and 941 cm^−1^, referring to the alkene C=C stretching vibration and the C=CH_2_ out-of-plane deformation vibration, respectively [12,37]. Furthermore, in comparing the TSC and MATSC spectra, a decrease in the absorption intensity that originated from the stretching vibration of the hydroxyl groups was observed at around 3487 cm^−1^ [12,30]. This additionally indicated the partial substitution of the hydroxyl groups with methacrylate moieties during the synthesis [12,30]. Analogously to this evaluation, the characteristic FTIR signals for further olefinic TSC derivatives can be found in the experimental section.

**Figure 3:**
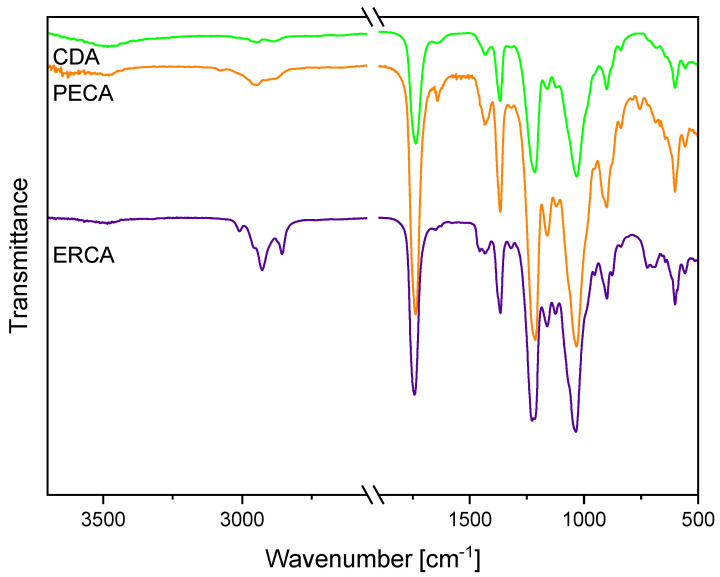
FTIR spectra of CDA, erucic CDA and 4-pentenoic CDA. The spectra have been shifted vertically for better readability.

The comparative FTIR characterization of the CDA and its photocrosslinkable derivatives 4-pentenoic CDA (PECA) and erucic CDA (ERCA) confirmed the successful esterification of the CDA with unsaturated carboxylic acids (Figure 3). PECA and ERCA were selected for detailed discussion because 4-pentenoic acid is short-chain and has a terminal carbon double bond while erucic acid is a long-chain fatty acid and exhibits a non-terminal carbon double bond. The educt CDA revealed a strong absorption band at 1738 cm^−1^, which was assigned to the carbonyl C=O ester stretching vibration [11,12,30]. Furthermore, significant vibrations of the CDA appeared at 1367 cm^−1^ and 1217 cm^−1^, corresponding to the methyl in-plane bending vibration and the C–O stretching vibration of the acetyl group, respectively [11,12,30]. After the functionalization of the CDA with 4-pentenoic acid, new absorption bands arose at 3084 cm^−1^, 1643 cm^−1^ and 758 cm^−1^, referring to the =CH_2_ stretching vibration, the alkene C=C stretching vibration and the =CH_2_ out-of-plane deformation vibration [11,38]. Accordingly, the modification of CDA with erucic acid resulted in additional absorption bands located at 3011 cm^−1^, 2924 cm^−1^, 2854 cm^−1^, 1651 cm^−1^, 953 cm^−1^, 878 cm^−1^ and 722 cm^−1^. The band at 3011 cm^−1^ was assigned to the =C–H stretching vibration of the carbon double bond of the unsaturated fatty erucic acid [39]. The distinctive absorption bands that appeared at 2924 cm^−1^ and 2854 cm^−1^ corresponded to the asymmetrical and symmetrical stretching of the aliphatic CH_2_ groups [39]. Moreover, the bands located at 1651 cm^−1^, 953 cm^−1^, 878 cm^−1^ and 722 cm^−1^ referred to the C=C stretching vibration and the –HC=CH– out-of-plane vibration of the erucic side group, respectively [39]. For both PECA and ERCA, the band for the C=O ester of the olefinic side group was not directly visible, since the absorption band of the acetyl ester, at around 1738 cm^−1^, overlapped with them.

Further in-depth structure determination of the biopolymers was achieved using ^13^C NMR spectroscopy (Table 1). The characteristic signals obtained from the ^13^C NMR measurement of the TSC revealed chemical shifts of 145.5 ppm, 132.8 ppm, 130.8 ppm and 128.3 ppm, which can, respectively, be assigned to the aromatic carbons of the substituted tosyl groups [31,35]. The signal at δ 21.7 corresponded to the methyl group of the tosylate [29,31]. Moreover, two signals could be detected at δ 69.2 ppm and 61.0 ppm, which indicated the presence of a tosyl group at the C6 position of the cellulose backbone or an unsubstituted C6 atom [29,31,35]. The peaks appearing at δ 166.5 ppm, 135.9 ppm, 126.2 ppm and 17.9 ppm confirmed the successful methacrylation of the TSC. The signal at δ 166.5 ppm corresponded to the carbonyl ester of the MATSC. Furthermore, the intensities at δ 135.9 ppm, 126.2 ppm and 17.9 ppm referred to the carbon double bond and the methyl group of the methacrylic moiety, respectively. The achieved degree of substitution (DS) after the tosylation of the microcrystalline cellulose was determined via elemental analysis (EA) and was defined as a DS_Tos_ of 0.85. However, the degree of the methacrylation could not be evaluated because the methacrylic side groups partially replaced the tosyl groups during the synthesis [31].

Characteristic chemical shifts in the CDA occurred as a duplet at 170.1 ppm and 169.3 ppm and referred to the acetate ester (Table 2). The signals at δ 20.0 ppm and 19.8 ppm represented the methyl groups of the acetate moieties [11,40,41]. The distinctive intensities from δ 100.4 to 62.3 ppm corresponded to the cellulose backbone [11,31]. After the functionalization of the CDA with 4-pentenoic acid, new signals arose at δ 172.1 ppm, 137.0 ppm, 115.4 ppm and 33.1 ppm. The carbonyl ester signal at δ 172.1 ppm confirmed the esterification reaction of the CDA with 4-pentenoic acid. Moreover, the intensities at δ 137.0 ppm and 115.4 ppm were assigned to the terminal carbon double bond. The peak at δ 33.1 ppm referred to the methylene group of 4-pentenoic acid, while the signal of the second CH_2_ group was superimposed by the solvent peak of the deuterated acetone at around 29 ppm. The ester signal at δ 172.7 ppm verified the functionalization of the CDA with the erucic acid [42,43]. Furthermore, the intensity at δ 129.8 ppm was assigned to the carbon double bond [42,43]. The peaks referring to the methyl group and the CH_2_ groups of the erucic moiety arose at δ 13.6 ppm and from 33.6 ppm to 22.5 ppm, respectively. Thus, the FTIR and NMR spectroscopy verified a successful functionalization of the TSC and the CDA with the olefinic side groups. Evaluation of the elemental composition obtained from the EA measurements resulted in a DS_acetate_ of 2.0 for the CDA. Based on that, a DS_pentenoic_ value of 0.6 and a DS_erucic_ value of 0.4 could be determined.

**Figure 4:**
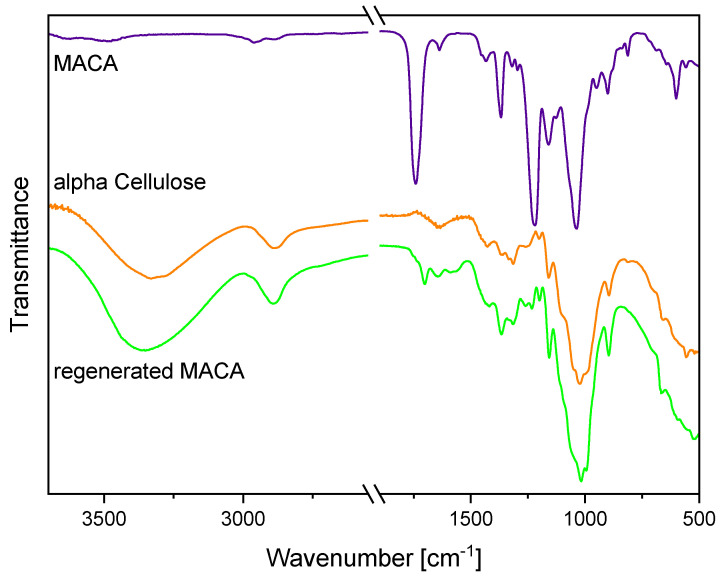
FTIR spectra of MACA, regenerated MACA and alpha cellulose. The spectra are shifted vertically for better readability.

After the regeneration of the photocrosslinked methacrylated CDA (MACA) with a sodium methoxide treatment, a FTIR analysis confirmed the reconversion of the MACA to cellulose (Figure 4). To ensure that the entire structure and not just the surface was regenerated into pure cellulose, a bulk structure was cut in half and FTIR measurements were carried out inside. Moreover, a comparison of the FTIR spectra of alpha cellulose to the regenerated MACA enabled validation of the achieved cellulose material. The FTIR spectrum of the MACA has already been described in former research papers [11,12] and is mainly characterized by absorption bands located at 1638 cm^−1^, 950 cm^−1^ and 813 cm^−1^, which correspond to the alkene C=C stretching vibration and the C=CH_2_ out-of-plane deformation vibration of the methacrylate group, respectively. After the regeneration process, the disappearances of the carbonyl C=O ester stretching vibration at 1738 cm^−1^ and the further characteristic absorption bands at 1638 cm^−1^, 1367 cm^−1^, 1217 cm^−1^, 950 cm^−1^ and 813 cm^−1^ indicated a successful reconversion from MACA to cellulose. Moreover, a significant broad absorption band arose at around 3352 cm^−1^; it was assigned to the stretching vibration of the hydroxyl groups [44]. Thus, this also implied the cleavage of the olefinic and acetate groups. Additionally, the comparison of the spectra of the regenerated MACA and the alpha cellulose led to the validation of the regeneration process due to their high level of agreement. It therefore seems entirely plausible that these cellulose derivatives are biodegradable after regeneration treatment.

### 2.2. Manufacturing of Cellulose-Based Architectures via Photocrosslinking and Subsequent Regeneration into Pure Cellulose

**Figure 5:**
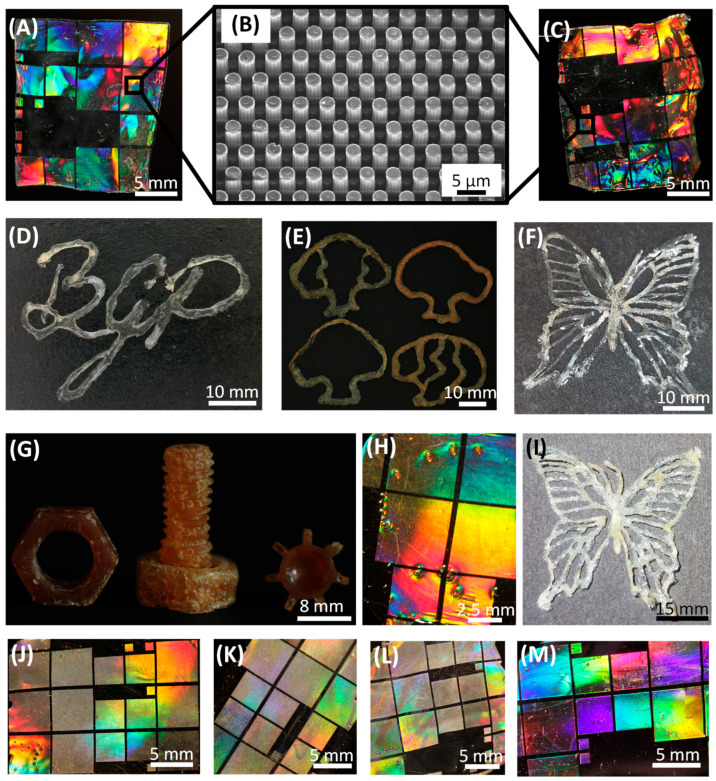
Architectures manufactured with cellulose-based photoresists. (**A**) Casted film (generated with methacrylated CDA) with an imprinted diffraction pattern that produced brilliant coloration when irradiated with white light. (**B**) The SEM image of the pattern, which consists of regularly aligned pillars in the micrometer range. (**C**) The pattern structure still revealed brilliant coloration after the film was regenerated into cellulose with sodium methoxide treatment, indicating that the macrostructure of the cellulose-based device was not significantly affected by the reconversion process. (**D**) A BGP logo made from the cellulose derivative MACA, written by hand with a pipette. (**E**) Various shapes of trees manually printed using the derivative MACA (according to the synthesis described in [12], which caused the yellowish to brownish coloration of the biopolymer). Each tree consists of five individual layers that were UV-cured before the next layer was printed. (**F**) A butterfly structure printed with the biopolymer MACA. (**G**) Various bulky parts, a nut, a screw and a gear, fabricated using layer-by-layer casting of the derivative MACA [12] into a PDMS mold. Each layer was photocrosslinked for 15 min. (**H**) Same as (**A**), but produced with a methacrylated TSC derivative. (**I**) A butterfly structure generated from MACA and entirely converted into cellulose with sodium methoxide. (**J**–**M**) Several casted films with imprinted diffraction patterns, produced by the photocrosslinking of various cellulose derivatives such as (**J**) oleic CDA, (**K**) linoleic CDA, (**L**) 4-pentenoic CDA and (**M**) sorbic CDA.

The photocrosslinkability of monomers or polymers is a prerequisite for their use in photoresists in additive manufacturing techniques such as direct laser writing [12,13,14], in other additive manufacturing processes or in general photochemical applications. The functionalization of CDA and TSC with olefinic side groups via esterification enabled a UV-induced photocrosslinking of these biopolymers. The created cellulose-based architectures were manufactured using layer-by-layer fabrication or casting in combination with imprinting (Figure 5).

MACA derivatives can be used for coatings or casted as films with imprinted patterns that display brilliant coloration when irradiated with white light (Figure 5A). For this purpose, a thin film of a MACA-based photoresist was casted onto a polydimethylsiloxane (PDMS) stamp, and during UV curing (15 min), the diffraction pattern was transferred into the cellulose-derivative-based film, which detached itself during crosslinking. The diffraction grating was composed of regularly arranged micropillars (Figure 5B). This procedure was also suitable for various olefinic CDA derivatives (Figure 5J–M) as well as for tosylated cellulose derivatives such as MATSC (Figure 5H), although these exhibited significantly longer solidification times due to the solvent used. In addition, objects consisting of multiple layers could be printed by hand using a pipette, with each layer being UV-cured before another layer is printed (Figure 5D–F). These specimens were also produced with MACA-based photoresists. However, the clearly recognizable color difference between (D) and (F) (transparent) and (E) and (G) (yellowish to brownish) resulted from different synthesis routes. The regions that appeared white in the otherwise transparent specimens resulted from air inclusions in the crosslinked biopolymers. Furthermore, it was also possible to produce various components from bulk material by casting the MACA-based photoresist layer-by-layer into a PDMS mold and subsequently UV-curing it (Figure 5G). The duration of UV curing for each individual layer was 15 min. Bio-based polymer derivatives are often not biodegradable due to their functional groups. To address this disadvantage, a regeneration process that converts olefinic cellulose derivatives into pure cellulose by employing sodium methoxide was developed in this research work. This regeneration treatment did not appear to affect the stability, the robustness or the overall shape of the manufactured objects (Figure 5C,I). Thus, the pattern structure of the converted cellulose film was still intact after the regeneration procedure and therefore revealed an identical brilliant coloration (Figure 5C) to that of the untreated MACA-based films. This indicates that the macrostructure of the cellulose-based architectures was not significantly damaged by the reconversion treatment. The biocompatible cellulose derivative MACA employed here is also suitable for the direct laser writing of 3D architectures in the submicrometer range [11,12,14]. Hence, these biopolymers could be employed for anticounterfeiting components, for tissue engineering devices or even as coatings for biologically inspired organic light-emitting diodes, along with the possible biodegradability of these parts after regeneration treatment [45,46].

## 3. Conclusions

A sustainable alternative to fossil-based polymers is the use of polysaccharides from renewable resources. The cellulose derivatives presented here can be photocrosslinked via UV curing. Therefore, cellulose diacetate and tosyl cellulose were functionalized with olefinic side groups via esterification in order to achieve photoreactive biopolymers, which are soluble in several organic solvents. Thus, they are suitable for various applications, e.g., crosslinkable coatings or photoresists for additive manufacturing techniques. Moreover, a procedure was developed for regenerating the manufactured cellulose-based devices entirely into pure cellulose architectures. Based on this transformation treatment, the biodegradability of these cellulose parts seems entirely plausible. This research work is an important step toward replacing petroleum-based polymers with biogenic ones, and, thus, it paves the way to expand the range of biomaterials for versatile UV-curing applications.

## 4. Experimental Section

Materials: All chemicals were used as received, without further purification.

Tosylation of the microcrystalline cellulose, adapted from [35]:

Microcrystalline cellulose, 10.25 g (Merck, Germany), and LiCl, 20.12 g (98%; VWR, Belgium), were dried in an oven at 105 °C overnight. The dried MCC was suspended in 200 mL of N,N-dimethylacetamide (DMAc, 99%; Carl Roth, Germany) and stirred at 130 °C for 2 h. After the suspension was allowed to cool to 100 °C, 20.12 g of ground LiCl was added. While the suspension was slowly cooled to room temperature under permanent stirring, the MCC dissolved completely. The obtained viscous cellulose solution revealed a yellowish–brownish coloration. In the next step, 200 mL of this MCC solution was cooled to 5 °C in an ice bath. A solution of 27.4 mL of triethylamine (99%; Acros Organics, France) and 13.5 mL of DMAC was added under permanent stirring. Afterward, a mixture of 18.01 g of p-toluenesulfonyl chloride (98%; Alfa Aesar, South Korea) and 20 mL of DMAc was added dropwise to the cellulose solution. This synthesis was allowed to react for 24 h at 5 °C. Then, the product was precipitated very slowly into 800 mL of ethanol (99.5%; Chemsolute, Germany). The precipitate was filtered off and washed with 200 mL of ethanol and 500 mL of distilled warm water (50 °C). Afterward, the product was dried by employing a rotary evaporator and via lyophilization.

Chemical functionalization of the tosylated cellulose:

Tosylated cellulose, 1.0 g, was dissolved in 40 mL of N,N-dimethylformamide (DMF, technical, VWR, France) under permanent stirring. Then, 1.5 mL of methacrylic acid anhydride (94%; Sigma-Aldrich, Germany) or, alternatively, another unsaturated carboxylic acid, 0.015 g of 4-dimethylaminopyridine (DMAP, 99%; Acros Organics, USA) and 2.1 g of dicyclohexylmethandiimine (DCC, 99%; Alfa Aesar, Germany) were added to the solution. The reaction was performed for 72 h at room temperature. The methacrylated TSC was precipitated in an excess of ethanol and washed with ethanol five times, with a centrifuge employed at 5 °C. After the evaporation of the ethanol, the product was received as a coarse powder.

3-butenoic TSC: 1.1 mL of 3-butenoic acid (96%; Alfa Aesar, UK); characteristic FTIR signals: 1738 cm^−1^ ν(C=O), 1643 cm^−1^ ν(C=C); and significant ^13^C NMR intensities (DMSO–d_6_, ppm): δ 171.3 (C=O) and 129.9 and 119.0 (C=C).

4-pentenoic TSC: 1.0 mL of 4-pentenoic acid (98%; Sigma-Aldrich, USA); FTIR: 1738 cm^−1^ ν(C=O), 1641 cm^−1^ ν(C=C); and ^13^C NMR (DMSO–d_6_, ppm): δ 172.6 (C=O) and 137.4 and 116.1 (C=C).

Sorbic TSC: 1.5 g of sorbic acid (99%; Alfa Aesar, USA); FTIR: 1723 cm^−1^ ν(C=O), 1645 cm^−1^ and 1615 cm^−1^ ν(C=C); and ^13^C NMR (DMSO–d_6_, ppm): δ 165.5 (C=O), 145.4, 139.9, 129.9 and 118.6 (C=C) and 18.1 (CH_3_).

Functionalization of CDA with olefinic side groups:

Cellulose diacetate, 1.2 g (pure; Carl Roth, Germany), was dissolved in 40 mL of dichloromethane (DCM, 98%; Alfa Aesar, Germany) under permanent stirring. Afterward, 2 mL of methacrylic acid anhydride or, alternatively, another unsaturated carboxylic acid, 0.015 g of DMAP and 2.1 g of DCC were added to the solution. The reaction was performed for 72 h at room temperature. The methacrylated CDA was precipitated in an excess of ethanol, while some of the other olefinic CDA derivatives were filtrated prior to precipitation. The product was washed with ethanol via centrifugation for five times. The derivative was obtained as a powder after the ethanol was evaporated. Additionally, CDA can be modified with methacrylic acid anhydride, according to the synthesis described in [12]. However, this synthetic route will lead to a slightly yellowish product.

3-butenoic CDA: 1.5 mL of 3-butenoic acid; characteristic FTIR signals: 3077 cm^−1^ ν(=C-H/=CH_2_), 1643 cm^−1^ ν(C=C) and 723 cm^−1^ δ(=CH_2_); and significant ^13^C NMR intensities (acetone–d_6_, ppm): δ 170.6 (C=O) and 130.8 and 118.2 (C=C).

4-pentenoic CDA: 3 mL of 4-pentenoic acid; FTIR: 3084 cm^−1^ ν(=C-H/=CH_2_), 1643 cm^−1^ ν(C=C) and 758 cm^−1^ δ(=CH_2_); and ^13^C NMR (acetone–d_6_, ppm): δ 172.1 (C=O) and 137.0 and 115.4 (C=C).

10-undecenoic CDA: 4.22 g of 10-undecenoic acid (99%; Alfa Aesar, USA); FTIR: 3078 cm^−1^ ν(=C-H/=CH_2_), 1642 cm^−1^ ν(C=C) and 722 cm^−1^ δ(=CH_2_); and ^13^C NMR (acetone–d_6_, ppm): δ 172.7 (C=O) and 139.1 and 113.9 (C=C).

Erucic CDA: 5.53 g of erucic acid (90%; Alfa Aesar, China); FTIR: 3011 cm^−1^ ν(=C-H), 2924 cm^−1^ and 2854 cm^−1^ ν(CH_2_), 1651 cm^−1^ ν(C=C) and 722 cm^−1^ δ(-HC=CH-); and ^13^C NMR (acetone–d_6_, ppm): δ 172.7 (C=O), 129.8 (C=C) and 13.6 (CH_3_).

Linoleic CDA: 5.2 mL of linoleic acid (95%; Alfa Aesar, Germany); FTIR: 3009 cm^−1^ ν(=C-H), 2929 cm^−1^ and 2857 cm^−1^ ν(CH_2_), 1653 cm^−1^ and 1626 cm^−1^ ν(C=C) and 721 cm^−1^ δ(-HC=CH-); and ^13^C NMR (acetone–d_6_, ppm): δ 172.7 (C=O), 129.9 (C=C), 128.0 (C=C) and 13.6 (CH_3_).

Oleic CDA: 5.5 mL of oleic acid (96%, VWR, France); FTIR: 3009 cm^−1^ ν(=C-H), 2927 cm^−1^ and 2852 cm^−1^ ν(CH_2_), 1626 cm^−1^ and 1575 cm^−1^ ν(C=C) and 723 cm^−1^ δ(-HC=CH-); and ^13^C NMR (acetone–d_6_, ppm): δ 172.9 (C=O), 129.7 and 128.0 (C=C) and 13.6 (CH_3_).

Sorbic CDA: 1.65 g of sorbic acid; FTIR: 1644 cm^−1^ and 1617 cm^−1^ ν(C=C), 801 cm^−1^ δ(C=CH); and ^13^C NMR (acetone–d_6_, ppm): δ 166.1 (C=O), 145.7, 140.3, 129.7 and 118.4 (C=C) and 18.1 (CH_3_).

Regeneration treatment:

For the regeneration process from a cellulose derivative to cellulose, a solution that consisted of sodium methoxide (98%, Alfa Aesar, Germany) and methanol (technical, VWR, France) was employed. Therefore, 0.1 g of sodium methoxide was slowly dissolved in 5 mL of methanol under stirring. The cellulose-derivative-based samples were immersed into this solution for 5 days at room temperature. Afterward, the cellulose specimens were washed with distilled water and acetone (technical, VWR, France). The samples were then dried via lyophilization to remove residual water.

Preparation of the photoresists for UV curing:

First, the CDA-based cellulose derivatives were dissolved in acetone and the olefinic TSC derivatives in DMSO (technical grade, VWR, France) under dark, ambient conditions and stirring. The olefinic CDA derivatives were dissolved in acetone, using seven parts of acetone by weight for one part of polymer, resulting in a honey-like viscosity. The viscosity of the photoresists could be adjusted based on the amount of solvent used, with a higher amount of solvent leading to a lower viscosity. The TSC derivatives were dissolved in DMSO, using three parts of DMSO by weight for one part of polymer. All acetone-based photoresists contained 2.5 wt.% of the photoinitiator 2-hydroxy-4′-(2-hydroxyethoxy)-2-methylpropiophenone (Irgacure 2959, 98%; Sigma-Aldrich, Italy) based on the weight of the polymer. Thus, all DMSO-based resists contained 2.5 wt.% of the photoinitiator 2-benzyl-2-(dimethylamino)-4′-morpholinobutyrophenone (Irgacure 369, 98%; BASF, Switzerland) based on the weight of the polymer. In each case, the photoresists were stirred for at least 30 min to ensure the homogeneous distribution of the photoinitiator. For initiatorless photocrosslinking based on the sorbic CDA, the preparation of the resist was carried out accordingly [11].

UV curing of the bio-based photoresists:

These photoresists could be UV-cured under a UV lamp (λ = 254 nm and λ = 365 nm; Herolab UV lamp 8 watts, Germany) in both liquid and solid states for at least 15 min. Suitable areas of application for these bio-based resists include coatings, film casting (also with imprinted patterns on the microscale), layer-by-layer casting of bulk structures or printing by hand. Therefore, the microstructures were imprinted on a micropatterned PDMS stamp during photocrosslinking. The bulk architectures were manufactured employing layer-by-layer casting in PDMS molds, with each layer being UV-cured. Manual printing was performed using pipettes or syringes with cannulas. Coatings were carried out according to the procedure described in [11].

Characterization:

Fourier transform infrared spectroscopy:

Fourier transform infrared spectra were received from the powders of the cellulose derivatives using a Nicolet 380 FTIR (Thermo Scientific, Germany) in attenuated total reflection mode. The spectra were recorded between 4000 and 500 cm^−1^, with a resolution of 4 cm^−1^ and 32 scans.

Nuclear magnetic resonance spectroscopy:

^13^C NMR spectra were recorded on a Jeol ECS-400 NMR (Japan) with at least 2048 scans. The evaluation was performed with Delta v 5.0.4 software (Jeol). The samples were analyzed as solutions in acetone–d6 or DMSO–d6 at 25 °C.

Elemental analysis:

The elemental composition of the cellulose derivatives was obtained by employing a Euro EA-Elemental Analyzer (Eurovector, Italy). Therefore, 1–3 mg of the powder of the biopolymer in tin crucibles was analyzed. This enabled the determination of the degree of substitution.

## Figures and Tables

**Table 1:** Peak assignments for the ^13^C NMR spectra of tosyl cellulose and methacrylated tosyl cellulose.

Sample	Assignment (ppm)							
	C=O (Meth-acrylic)	C=C (Aromatic)	C=C (Meth-acrylic)	C1–C5 (Backbone)	C6 (Tosylated)	C6 (Non-Tosylated)	CH3 (TS)	CH3 (Meth-acrylic)
TSC	–	145.5 132.8 130.8 128.3	–	102.6–72.3	69.2	61.0	21.7	–
MATSC	166.5	145.5 132.9 130.7 128.1	135.9 126.2	100.0–72.4	–	61.5	21.6	17.9

**Table 2:** Peak assignments for the ^13^C NMR spectra of CDA [11], 4-pentenoic CDA and erucic CDA.

Sample	Assignment (ppm)						
	C=O (Acetate)	C=O (Olefinic)	C=C (Olefinic)	C1–C6 (Backbone)	CH_2_ (Olefinic)	CH_3_ (Acetate)	CH_3_ (Olefinic)
CDA	170.1 169.3	–	–	100.4–62.3	–	20.0 19.8	–
PECA	170.1 169.3	172.1	137.0 115.4	100.4–62.3	33.1	20.0 19.8	–
ERCA	170.1 169.2	172.7	129.8	100.4–62.3	33.6–22.5	20.0 19.8	13.6

## Data Availability

Supporting information is available from the authors upon request.

## References

[1] Wassmann P. (2011). Arctic marine ecosystems in an era of rapid climate change. Prog. Oceanogr..

[2] Wells M.L., Karlson B., Wulff A., Kudela R., Trick C., Asnaghi V., Berdalet E., Cochlan W., Davidson K., De Rijcke M. (2020). Future HAB science: Directions and challenges in a changing climate. Harmful Algae.

[3] Abbass K., Qasim M.Z., Song H., Murshed M., Mahmood H., Younis I. (2022). A review of the global climate change impacts, adaptation, and sustainable mitigation measures. Environ. Sci. Pollut. Res..

[4] Roode-Gutzmer Q.I., Kaiser D., Bertau M. (2019). Renewable methanol synthesis. ChemBioEng Rev..

[5] Rothammer B., Neusser K., Marian M., Bartz M., Krauß S., Böhm T., Thiele S., Benoit M., Detsch R., Wartzack S. (2021). Amorphous carbon coatings for total knee replacements—Part i: Deposition, Cytocompatibility, chemical and mechanical properties. Polymers.

[6] Klemm D., Heublein B., Fink H.P., Bohn A. (2005). Cellulose: Fascinating biopolymer and sustainable raw material. Angew. Chem. Int. Ed..

[7] Rothammer M., Zollfrank C., Busch K., von Freymann G. (2021). Tailored disorder in photonics: Learning from nature. Adv. Opt. Mater..

[8] Reimer M., Eckel F., Rothammer M., Van Opdenbosch D., Zollfrank C. (2023). Manufacturing of cellulose-based nano- and submicronparticles via different precipitation methods. Cellulose.

[9] Wang J., Chen J., Wang W., Lu J., Li Y., Bai T., Chen J., Zhu Z., Wang D. (2023). Preparation of boron-containing chitosan derivative and its application as intumescent flame retardant for epoxy resin. Cellulose.

[10] Wang S., Lu A., Zhang L. (2016). Recent advances in regenerated cellulose materials. Prog. Polym. Sci..

[11] Rothammer M., Strobel P., Zollfrank C., Urmann C. (2023). Biocompatible coatings based on photo-crosslinkable cellulose derivatives. Int. J. Biol. Macromol..

[12] Rothammer M., Heep M.C., von Freymann G., Zollfrank C. (2018). Enabling direct laser writing of cellulose-based submicron architectures. Cellulose.

[13] Meiers D.T., Rothammer M., Maier M., Zollfrank C., von Freymann G. (2023). Utilizing the Sensitization Effect for Direct Laser Writing in a Novel Photoresist Based on the Chitin Monomer N-acetyl-D-glucosamine. Adv. Eng. Mater..

[14] Rothammer M., Meiers D.T., Maier M., Von Freymann G., Zollfrank C. (2023). Initiator-free photo-cross-linkable cellulose-based resists for fabricating submicron patterns via direct laser writing. JOSA B.

[15] Nobbs J.D., Zainal N.Z., Tan J., Drent E., Stubbs L.P., Li C., Lim S.C., Kumbang D.G., van Meurs M. (2016). Bio–based Pentenoic Acids as Intermediates to Higher Value-Added Mono-and Dicarboxylic Acids. ChemistrySelect.

[16] Parodi A., Jorea A., Fagnoni M., Ravelli D., Samorì C., Torri C., Galletti P. (2021). Bio-based crotonic acid from polyhydroxybutyrate: Synthesis and photocatalyzed hydroacylation. Green Chem..

[17] Kindler A., Zelder O. (2022). Biotechnological and Chemical Production of Monomers from Renewable Raw Materials. Advances in Polymer Science.

[18] Huang S.H., Liu P., Mokasdar A., Hou L. (2013). Additive manufacturing and its societal impact: A literature review. Int. J. Adv. Manuf. Technol..

[19] Marian M., Zambrano D.F., Rothammer B., Waltenberger V., Boidi G., Krapf A., Benoit M., Stampfl J., Rosenkranz A., Gachot C. (2023). Combining multi-scale surface texturing and DLC coatings for improved tribological performance of 3D printed polymers. Surf. Coat. Technol..

[20] Schroeter J., Felix F. (2005). Melting cellulose. Cellulose.

[21] Ganster J., Fink H.P. (2013). Cellulose and cellulose acetate. Bio-Based Plastics: Materials and Applications.

[22] Rees A., Powell L.C., Chinga-Carrasco G., Gethin D.T., Syverud K., Hill K.E., Thomas D.W. (2015). 3D bioprinting of carboxymethylated-periodate oxidized nanocellulose constructs for wound dressing applications. BioMed Res. Int..

[23] Pattinson S.W., Hart A.J. (2017). Additive manufacturing of cellulosic materials with robust mechanics and antimicrobial functionality. Adv. Mater. Technol..

[24] Melilli G., Carmagnola I., Tonda-Turo C., Pirri F., Ciardelli G., Sangermano M., Hakkarainen M., Chiappone A. (2020). DLP 3D printing meets lignocellulosic biopolymers: Carboxymethyl cellulose inks for 3D biocompatible hydrogels. Polymers.

[25] Chan C.L.C., Lei I.M., van de Kerkhof G.T., Parker R.M., Richards K.D., Evans R.C., Huang Y.Y.S., Vignolini S. (2022). 3D printing of liquid crystalline hydroxypropyl cellulose—Toward tunable and sustainable volumetric photonic structures. Adv. Funct. Mater..

[26] Rozicka A., Niemistö J., Keiski R.L., Kujawski W. (2014). Apparent and intrinsic properties of commercial PDMS based membranes in pervaporative removal of acetone, butanol and ethanol from binary aqueous mixtures. J. Membr. Sci..

[27] Heinze T., Liebert T. (2001). Unconventional methods in cellulose functionalization. Prog. Polym. Sci..

[28] Tischer T., Goldmann A.S., Linkert K., Trouillet V., Börner H.G., Barner-Kowollik C. (2012). Modular ligation of thioamide functional peptides onto solid cellulose substrates. Adv. Funct. Mater..

[29] Schmidt S., Liebert T., Heinze T. (2014). Synthesis of soluble cellulose tosylates in an eco-friendly medium. Green Chem..

[30] Barud H.S., Júnior A.M.d.A., Santos D.B., de Assunção R.M., Meireles C.S., Cerqueira D.A., Filho G.R., Ribeiro C.A., Messaddeq Y., Ribeiro S.J. (2008). Thermal behavior of cellulose acetate produced from homogeneous acetylation of bacterial cellulose. Thermochim. Acta.

[31] El-Sayed N.S., Abd El-Aziz M.E., Kamel S., Turky G. (2018). Synthesis and characterization of polyaniline/tosylcellulose stearate composites as promising semiconducting materials. Synth. Met..

[32] Takacs E., Wojnarovits L., Földváry C., Borsa J., Sajó I. (2001). Radiation activation of cotton-cellulose prior to alkali treatment. Res. Chem. Intermed..

[33] Liu C., Baumann H. (2005). New 6-butylamino-6-deoxycellulose and 6-deoxy-6-pyridiniumcellulose derivatives with highest regioselectivity and completeness of reaction. Carbohydr. Res..

[34] Cabassi F., Casu B., Perlin A.S. (1978). Infrared absorption and Raman scattering of sulfate groups of heparin and related glycosaminoglycans in aqueous solution. Carbohydr. Res..

[35] Rahn K., Diamantoglou M., Klemm D., Berghmans H., Heinze T. (1996). Homogeneous synthesis of cellulose p-toluenesulfonates in N, N-dimethylacetamide/LiCl solvent system. Die Angew. Makromol. Chem. Appl. Macromol. Chem. Phys..

[36] Freeman D., Hamble A.N. (1957). Spectra of sulphonyl derivatives. IV. Sulphonic esters. Aust. J. Chem..

[37] Träskman B., Tammela V. (1986). The preparation and properties of vinyl cellulose. J. Appl. Polym. Sci..

[38] Valentin H.E., Berger P.A., Gruys K.J., Rodrigues M.F.d.A., Steinbüchel A., Tran M., Asrar J. (1999). Biosynthesis and characterization of poly (3-hydroxy-4-pentenoic acid). Macromolecules.

[39] Sherazi S.T.H., Arain S., Mahesar S.A., Bhanger M.I., Khaskheli A.R. (2013). Erucic acid evaluation in rapeseed and canola oil by Fourier transform-infrared spectroscopy. Eur. J. Lipid Sci. Technol..

[40] Kono H. (2013). Chemical shift assignment of the complicated monomers comprising cellulose acetate by two-dimensional NMR spectroscopy. Carbohydr. Res..

[41] Kono H., Hashimoto H., Shimizu Y. (2015). NMR characterization of cellulose acetate: Chemical shift assignments, substituent effects, and chemical shift additivity. Carbohydr. Polym..

[42] Kok W.M., Mainal A., Chuah C.H., Cheng S.F. (2018). Content of erucic acid in edible oils and mustard by quantitative 13C NMR. Eur. J. Lipid Sci. Technol..

[43] Zhang Y., Mu M., Yang Z., Liu X. (2022). Ultralong-chain ionic liquid surfactants derived from natural erucic acid. ACS Sustain. Chem. Eng..

[44] Kunusa W.R., Isa I., Laliyo L.A., Iyabu H. (2018). FTIR, XRD and SEM analysis of microcrystalline cellulose (MCC) fibers from corncorbs in alkaline treatment. Proceedings of the 2nd International Conference on Statistics, Mathematics, Teaching, and Research.

[45] Kim J.J., Lee J., Yang S.P., Kim H.G., Kweon H.S., Yoo S., Jeong K.H. (2016). Biologically inspired organic light-emitting diodes. Nano Lett..

[46] Zyla G., Kovalev A., Heisterkamp S., Esen C., Gurevich E.L., Gorb S., Ostendorf A. (2019). Biomimetic structural coloration with tunable degree of angle-independence generated by two-photon polymerization. Opt. Mater. Express.

